# Thermo-Mechanical Characterization of 4D-Printed Biodegradable Shape-Memory Scaffolds Using Four-Axis 3D-Printing System

**DOI:** 10.3390/ma16145186

**Published:** 2023-07-24

**Authors:** Vukasin Slavkovic, Nikola Palic, Strahinja Milenkovic, Fatima Zivic, Nenad Grujovic

**Affiliations:** Faculty of Engineering, University of Kragujevac, 34000 Kragujevac, Serbia; vukasin@fink.rs (V.S.); palic@fink.rs (N.P.); strahinja.milenkovic@fink.rs (S.M.); gruja@kg.ac.rs (N.G.)

**Keywords:** additive manufacturing (AM), FDM 3D printing, 4D printing, smart materials (SM), thermo-mechanical testing, shape-memory materials (SMMs), biomedical devices, biodegradable vascular stents (BVS), material extrusion (MEX)

## Abstract

This study was conducted on different models of biodegradable SMP (shape-memory polymer) scaffolds. A comparison was conducted utilizing a basic FDM (fused deposition modeling)/MEX (material extrusion) printer with a standard printing technique and a novel, modified, four-axis printing method with a PLA (poly lactic acid) polymer as the printing material. This way of making the 4D-printed BVS (biodegradable vascular stent) made it possible to achieve high-quality surfaces due to the difference in printing directions and improved mechanical properties—tensile testing showed a doubling in the elongation at break when using the four-axis-printed specimen compared to the regular printing, of 8.15 mm and 3.92 mm, respectfully. Furthermore, the supports created using this method exhibited a significant level of shape recovery following thermomechanical programming. In order to test the shape-memory effect, after the thermomechanical programming, two approaches were applied: one approach was to heat up the specimen after unloading it inside temperature chamber, and the other was to heat it in a warm bath. Both approaches led to an average recovery of the original height of 99.7%, while the in-chamber recovery time was longer (120 s) than the warm-bath recovery (~3 s) due to the more direct specimen heating in the latter case. This shows that 4D printing using the newly proposed four-axis printing is an effective, promising technique that can be used in the future to make biodegradable structures from SMP.

## 1. Introduction

The additive manufacturing (AM) process, known as fused deposition modeling (FDM) [1,2], fused filament fabrication (FFF) or, according to ISO/ASTM 52900, material extrusion (MEX) [3,4], is widely used and may be found in both open-source/cheap and industrial/high-end AM systems. For the purpose of creating heterogeneous components using FDM/FFF, the standard AM-production process is frequently used [1,2,3,5,6]. In general, it can be stated that there are four widely used technologies of 3D printing—fused deposition modeling (FDM), vat polymerization [4], stereolithography (SLA) [7], and the digital light process (DLP) [8]. The process of printing begins with the construction of 3D models that immediately produce the appropriate geometrical form and structure using CAD software, such as Catia V5 R21 software. The necessary slicing and route-planning software uses the geometrical data from these models to produce machine-instruction files for the FDM/FFF system. Following the creation of the components using the FDM/FFF technique, they are post-processed and finished in accordance with the application [9,10]. Along with the development of additive technologies, there is also the development of printing materials, such as PLA [2], PEEK [11], resin [12,13] etc.

Smart materials (SMs) can be defined as materials which change their behavior as a response to a specific stimuli, such as the alteration of magnetic or electric fields, stress, temperature, or chemical properties [14,15]. These materials can be produced by additive technologies but, to deal with material characteristics’ evolution over time, a new term, “4D printing,” was introduced (3D printing + time) [16]. This is a method for designing complex structures with the ability to change over time in response to external stimuli, including changes in the shape, structure, or function of 3D printing over time [16,17,18].

Thermo-responsive shape-memory materials (SMMs) are the most acclaimed type of SMM utilized in 3D/4D printing [19]. Shape-memory effects can be divided into the following types: two-way shape-memory effects—materials that can memorize only one shape; and three-way shape-memory effects—materials that can memorize more than one shape [20]. Shape-memory polymers, in comparison with shape-memory alloys, can exhibit deformations exceeding 400%, which allows them to be applied in biomedicine, microsystem actuators, aerospace structures, etc. [15,21,22,23,24,25].

The development of modern printers enables the fabrication of thermo-activatable cardiovascular stents [26], supports for tissue cultures [27,28] drug-delivery devices [29], elastomeric materials [30], gastroretentive drug-delivery systems [31], and others.

The material must undergo a thermomechanical loading-and-unloading cycle to develop a thermally induced SMP device. By applying a mechanical load at a high temperature, T_H_, the SMP is first deformed (isothermally programmed) from an initially undeformed shape. After the temperature is lowered to T_L_ and the external mechanical load is removed, the material retains its deformed shape, called the temporary or programmed shape. If the temperature does not change, the shape is usually maintained. If the temperature is increased to T_D_, the shape-memory phenomenon is triggered, and the initial shape is recovered. In general, T_H_ and T_D_ are above the glass-transition temperature T_g_, and T_L_ is below T_g_ [15].

Poly lactic acid (PLA) is a leading biomaterial in numerous applications in industry, replacing conventional polymers, as well as in medicine, due to its biocompatibility and biodegradability, hydrophobicity, and good mechanical and physical properties. Furthermore, PLA is also a Food and Drug Administration (FDA)-approved material [32] for use in food packaging [33], oral medicine [34] and the production of stents [35].

In addition, PLA exhibits shape-memory characteristics. Shape-memory polymers (SMPs) are polymeric smart materials with the ability to recover from a deformed state to their original state when exposed to external stimuli [26]. The utilization of this effect in cardiovascular stent procedures can reduce the catheter size for delivery and, thus, reduce the wound size, allowing faster recovery. Stents made of shape-memory polymers can also guarantee self-expansion at body temperature, excluding auxiliary devices, for these operations, while allowing storage at room temperature [36]. Beyond PLA [37], the most widely used polymers in 4D printing also include PETG [38], Clear [39,40], polypropylene [3,5,6], and others.

In order to restore normal blood flow after the medical treatment of clogged vessels, scaffold implementation is an essential procedure to widen arteries and allow vessel remodeling. Bare metal stents (BMS) are the most typically utilized types of stent, but they have disadvantages. The corrosion of BMS can lead to atherosclerosis within the vessel [41]. In addition, the metallic toxic ions released from the stent can trigger an inflammatory response [42]. If this occurs, the repetition of the procedure to replace or remove the stent is inevitable, which is in itself stressful for patients and carries certain risks [10]. Novel studies have found alternative, bioabsorbable and biodegradable vascular stents (BVS) due to their ability to provide support over a predefined amount of time, before they are reabsorbed within the vessel, removing the need for further surgery [43]. In general, PLA based stents have received a great deal of attention, due to being fully biocompatible for human use [10,35,44,45,46]. As previously stated, the main BVS advantage over BMS is that it does not corrode, whereas BMS corrosion can further provoke atherosclerosis and potential inflammatory response due to toxicity of the metallic ions. Additional BVS advantage is that during its dissolving, endothelial function is restored, thus helping artery to return to its physiological role (both systolic and diastolic) [47,48]. The first polymer BVS generation were initially made of poly-L-lactic (PLLA): Igaki-Tamai [48], ABSORB and DESolve scaffolds [49]. Subsequently, stent varieties with enhanced properties and novel features have been studied aiming at reduced material consumption and lower strut thickness in order to minimize the blood flow interaction and narrowing of the vessel lumen [47,50]. The latest polymer-based BVS devices are made of PLLA and its derivates [35,47,48,49,50]. New generation BVS devices showed certain disadvantages, such as difficult balance between reabsorption rate and mechanical vessel support [51] Although stent biodegradability can provide resuming of a native coronary functions it still remains novel research field and BMS still remains primary choice in a majority of cases [47,48]. The next generation BVSs are expected to further improve and to overcome current drawbacks [47].

Some of the most used geometrical models for BVS in literature are: Monorail [35], Absorb [52], Racetrack [53], Curved diamond, Diamond and Skewed parallelogram [53,54], which is chosen for our study.

Biodegradable vascular scaffolds, as radially loaded structures, bear load on their walls, and their improvement can achieved in layer-by-layer printing, in a cylindrical coordinate system. For this purpose, we have created a special four-axis printing system with a similar concept as in [37,46,55]. As the structure of the biodegradable stents mainly assumes cylindrical supporting structures, the standard layer-by-layer manufacturing method did not provide adequate mechanical characteristics, except in the case of test prototypes [53]. The standard printer has been rearranged to enable rotational printing around the three orthogonal X, Y, Z axes, beside the linear printing along these X, Y, Z axes. In this way, the melted filament is applied directly on the rotating barrel without supports. The resulting structure has significantly higher reliability, better mechanical response, and better surface quality. Also, the radial load now acts on the radially applied material layers, which improves the mechanical response of the material.

To the best of our knowledge, although specific elements has been used, there is no study where all of these elements are jointly applied to create the scaffolds by using four-axis printing [37,46,55] of smart materials through the 4D printing process. In this paper, a novel method of printing and testing of the 4D printed PLA biomedical devices is presented, in particular related to the biodegradable vascular stents (BVS).

The main goal of this paper is to test the new methods of printing BVS by using 4D printing. The influence of the BVS structure on its behavior in the process of shape recovery was examined. Two methods of thermal activation of the shape memory PLA BVS were used in the shape recovery test: (i) direct recovery in a hot bath and (ii) heating the sample after thermo-mechanical testing in a temperature chamber. The main novelty and improvement presented in this paper is the acceleration of the BVS four-axis 4D printing and testing process, which reduces the production time from concept to manufacturing, and improves the quality and precision of the complex cylindrical structures such as BVS. Also, the new printing method with four-axis printing reduces the number of failures, as commonly occuringwith conventional vertical layer-by-layer printing.

## 2. Materials and Methods

In this paper, authors used two FDM 3D printing methods in order to compare production quality of printed BVSs. Furthermore, microscopic surface examination of the samples obtained by both methods was compared. At last, shape memory effect of the printed BVSs was examined.

### 2.1. Materials and 3D Printing

**Figure 1:**
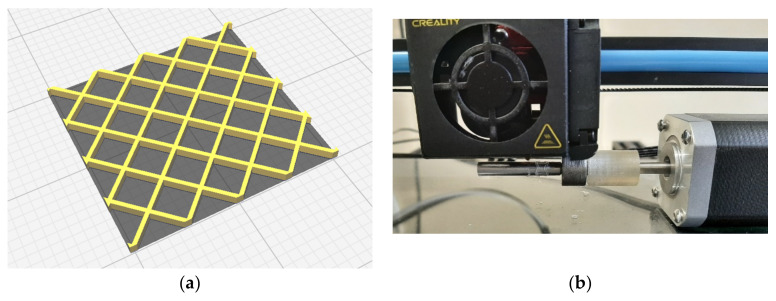
Unfolded CAD design (**a**) and four-axis printing method (**b**).

Commercial Creality CR-10S5 FDM printer with 0.4 mm nozzle was used to print the PLA scaffold sample. All scaffold samples are printed by using 100% infill density, 0.1 mm layer thickness for the standard printing and 0.4 mm layer thickness for the four-axis printing. Both prints were realised without a bed/drum heating. The printing speed was set at 10 mm/s, with nozzle temperature of 230 °C. Unfolded structure CAD model preparation with the 3D printer slicer software, and 3D printing on the rotating drum are showed in Figure 1.

**Figure 2:**
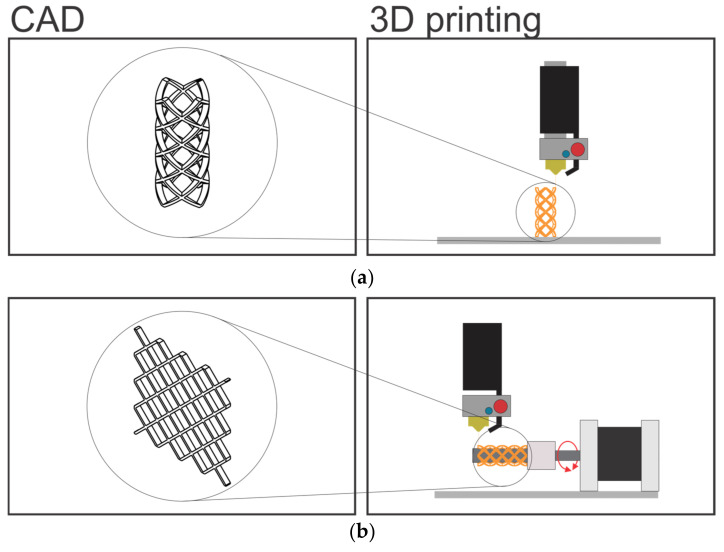
Different CAD model preparation (**left**) for different printing technologies (**right**): (**a**) CAD model (**left**) for the standard FDM printing (**right**); (**b**) Unfolded CAD model (**left**) for the rotational print on the cylindrical form (**right**).

CAD models are prepared, in Catia V5 R21 software, with two approaches for the two printing methods. For a standard FDM technology, CAD model of the final sample geometry is prepared and for the four-axis printing, the CAD model is prepared as the unfolded form, as shown in Figure 2.

The printer nozzle temperature was 230 °C. In this research a transparent PLA filament from Devil Design of 1.75 diameter was used as the raw material for FDM 3D printing. Printing parameters of both methods are very similar. The same nozzle orifice diameter was used for both printing methods. A low printing speed of 10 mm/s was used in order to provide appropriate printing accuracy for a very thin ligaments. Adhesion of the first layer on the printer’s bed at standard FDM printing was achieved by introducing a raft in order to increase the contact surface of the printed object to the printing bed and to prevent debonding. The layer thickness for four-axis printing is 0.4 mm because the complete geometry is printed on the drum during one cycle so that the ligaments remain as homogeneous as possible. The printing parameters are represented in Table 1. In order to avoid printing defects, filament was dried in vacuum chamber at 60 °C for 24 h, prior to the printing.

**Figure 3:**
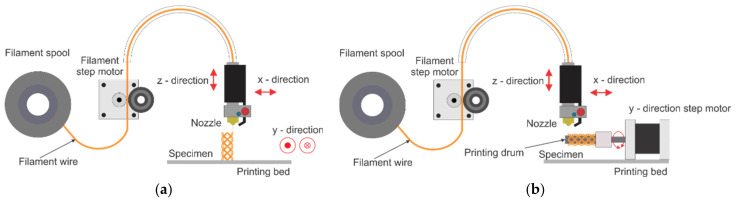
A schematic presentation of the typical FDM printing process (**a**) and the proposed four-axis printing (**b**). Bullet and fork (y-direction in the left image) denote reciprocating motion in two orientations of the y axis.

The schematic presentations of the two types of FDM printing are shown in Figure 3.

By including rotating axis, in addition to the original X-Y-Z axes, a four-axis printing system is created. In this way the stents can be printed directly on the rotating drum without supporting structures, as shown in Figure 3b. The four-axis printing method thereby enhances surface integrity while also improving manufacturing efficiency. The Y-axis translatory movement is translated into the drum rotary movement, thus avoiding the need for a printer reprogramming or software changes. The drum used for this experiment has the diameter of 6 mm, while the Y-axis gear outer diameter is 12 mm, which corresponds to the 2:1 ratio. Therefore, 10 mm/s setting in Y direction corresponds to the drum angular speed of 1.57 rad/s.

In order to show the improvement in the quality of the final printed objects, the traditional FDM and newly proposed four-axis printing methods were compared. The schematic representations of these procedures are shown in Figure 3. Dimensions of the printed BVS were: length of 20.5 mm, inner diameter of 6.2 mm, outer diameter of 7 mm, ligament thickness of 0.5 mm and ligament width of 0.4 mm, as shown in Table 2.

Optical microscopy of the samples was done by using optical microscope BTC STM-8T Trinocular Stereo Microscope, Budapest Telescope Center, Budapest, Hungary, with separated upper and lower LED illumination and a Touptek Photonics (Budapest Telescope Center, Budapest, Hungary), digital camera UCMOS03100KPA (3.1 MP, 1/2“sensor) attached.

### 2.2. Uniaxial Thermo-Mechanical Characterization of BVS

**Figure 4:**
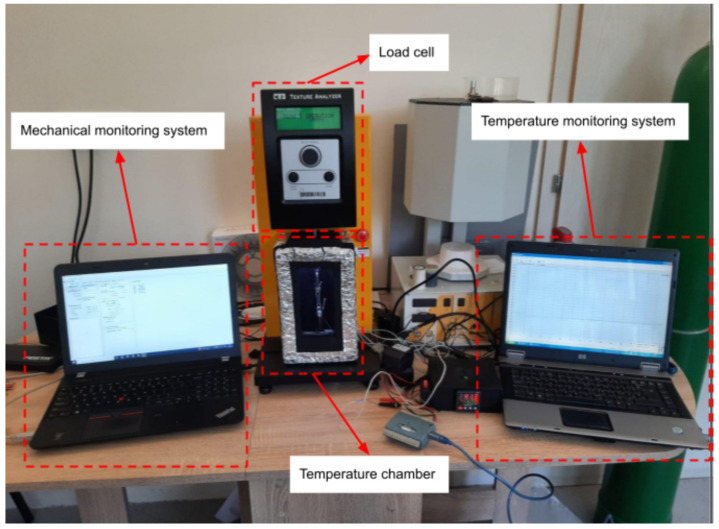
Laboratory measurement system.

Uniaxial thermo-mechanical and tensile tests were performed by using Brookfield CT3 Texture Analyzer equipped with the in-house built thermal chamber. The instrument has a calibrated load cell of 500 N maximum load and with 0.05 N precision and capability to work as a standalone system and as a live monitoring system. Heating chamber with its own temperature monitoring system was developed for this experiment (Figure 4).

The temperature chamber has its own heating and cooling characteristics. In the case of the tests where samples were heated within this chamber, periods of heating to a predefined temperature and periods of its cooling down to the room temperatures, has also been considered. Hence, chamber temperature properties have significant influence on the results. The temperature cycles were: heating from the room temperature (approx. 25 °C) to the temperature of 70 °C, then holding it for 10 min (600 s), after which it is cooled down to the room temperature. Duration of one such temperature cycle was approximately 900 s. The resolution of a temperature measurement was 0.1 °C.

### 2.3. Shape-Memory Characterization of BVS—Griping Technique and Temperature Chamber Control, Recovery in Temperature Chamber, Recovery in Hot Bath

The scaffolds were examined by the thermomechanical cycle (TMC) and the mechanical behavior of the structures in such conditions was monitored. The printed scaffolds were placed in the initial position on the tension jaws, then the temperature of the chamber was uniformly increased to the 70 °C. When the chamber reaches the target temperature, it is maintained for a certain period of time (e.g., 10 min), in order to uniformly heat the entire volume of the sample. The straining began at a speed of 0.5 mm/s up to a maximum displacement of 10 mm. The strained sample was cooled in the chamber to the temperature of 25 °C and the loads were monitored that are generated by the internal stresses due to the cooling. When the sample completely cools down, it is unloaded from one jaw and heated again in the chamber, and the change in the sample geometry generated by the temperature increase was also monitored.

The shape recovery process was conducted by using the two different approaches: sample heating in a warm bath at 70 °C and by heating the samples up to 70 °C in the temperature chamber.

BVS was thermomechanically programmed by applying uniaxial tensile deformation at 70 °C, when the structure was held at that temperature for 10 min, after which it began to cool down, to the room temperature. Then, the load was removed, and scaffolds were held in a deformed state. The printed BVSs were programmed in full thermo-mechanical cycle as follows:

- specimen constraining
- heating from the room temperature up to T_h_ = 70 °C
- uniaxial loading—at 0.5 mm/s until total displacement of 10 mm is reached
- constrained cooling back to the room temperature
- unloading
- heating over T_g_ for the full shape recovery

For a hot bath shape recovery, transparent container with hot water was used. The shape recovery process was captured by the camera. The temperature of the water was 70 °C and the printed scaffold was kept in the bath for at least 60 s in order to ensure full shape recovery process.

The programming parameters for the shape recovery test were selected according to the technical capabilities of the measuring equipment—the test machine and the in-house built temperature chamber. As the behavior of the PLA depends on both the rate of deformation and the temperature, our investigation initially focused on the selection of these two parameters. The test machine allows a maximum speed of 1 mm/s during tension and a value of 50% of the nominal maximum speed was selected. When choosing the programming temperature, the selection was based partialy on previous experience and also based on the results from the literature [37] which show that the glass transition temperature of PLA is approx. 60 °C. The selection of a programming temperature of 70 °C enabled effective tensile testing within the flexible “rubbery” state of the material, as well as the optimal heating time until the activation of shape recovery was achieved.

## 3. Results and Discussion

### 3.1. Microscopy Investigations

**Figure 5:**
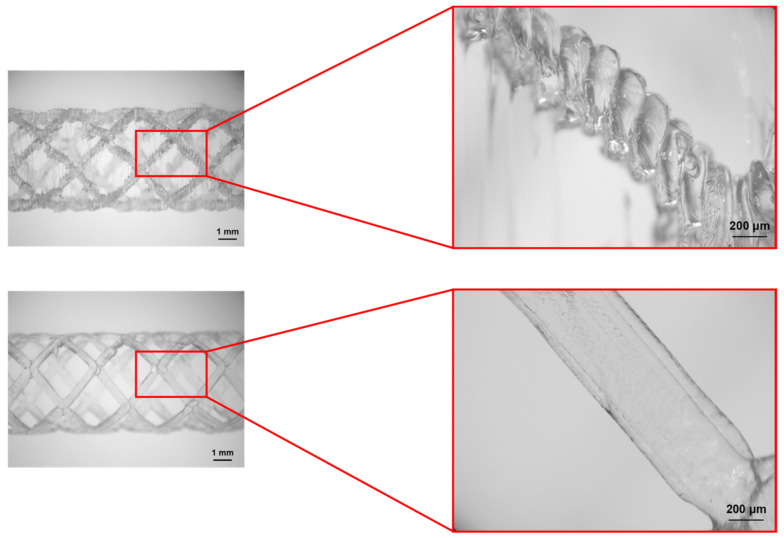
Magnified view of one BVS ligament—standard 3D printing (**upper**) and four-axis printing (**lower**).

Figure 5 shows microscopic images of the BVS printed by using two different methods. The scaffolds’ dimensions are listed in Table 2. Based on the results, it is clear that the four-axis printed BVS outperformed the standard print method in terms of the surface quality. Four-axis method produced surfaces with smooth and rounded edges at BVS. These characteristics are very beneficial since they will reduce the stress concentration on vascular tissue, as well as any interference with a blood flow. Hence, the risk of inflammation is decreased and the vessel healing process is promoted. It has been observed that the sharp edges of stents produced via the traditional FDM process can cause damage to the vessels. Additionally, it can cause stress concentration within the structure that can have negative effects on the patient vessels [46]. It was shown that FDM printed parts exhibit superior tribological characteristics along the fiber printing direction when compared both to direction transverse to a printed fibers direction and to a layer stacking direction [56].

From the Figure 5, it is clear that the durability of a stent created by the traditional printing method strongly depends on the strength of the layers’ adhesion, particularly when the radius is reduced due to the uniaxial loading. On the other hand, the applied axial load on the stents printed by the novel four-axis printing method acts perpendicularly to the plane of material deposition which makes it stronger and more reliable.

### 3.2. Tensile-Testing of BVS

**Figure 6:**
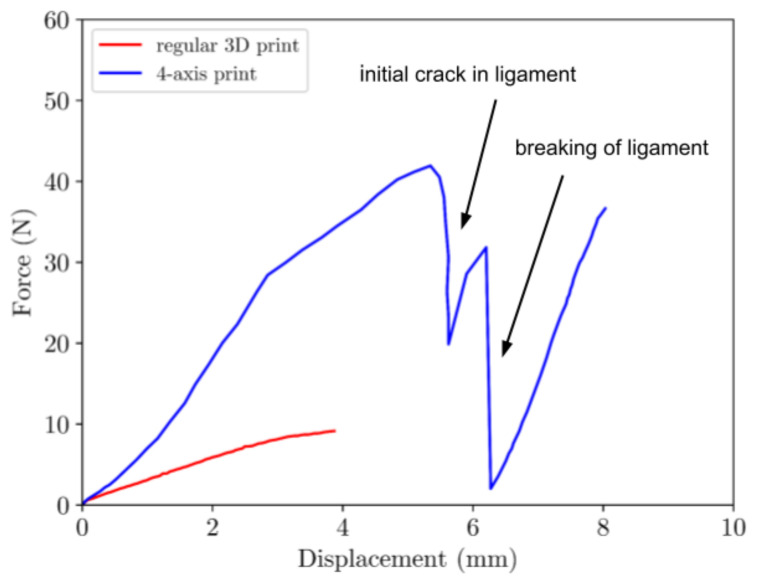
Tensile properties of the two types of samples—red line represents traditional 3D printing; blue line represents four-axis 4D printing.

In order to evaluate the potential improvement of the mechanical characteristics resulting from the new printing method, we conducted tensile tests on both sample types at standard room temperature. The mechanical behavior of both types of samples is determined by the brittle PLA nature at temperatures lower than T_g_. Results given in Figure 6 show that the mechanical properties of the samples produced by the four-axis printing were significantly better than of those made by traditional printing. The elongation at break of the samples produced by the four-axis 4D printing is significantly higher: 8.15 mm compared to 3.92 mm for traditionally printed samples. In the case of four-axis printing, the structural integrity of the printed scaffold undergoes a three-stage process of breaking. Initially, there is a load decrease due to the crack initiation in one of the ligaments. Subsequently, a significant load decline appears upon the complete rupture of the ligament. Ultimately, the entire structure collapses during the third and final stage. The new method of printing not only increases their elongation but also significantly improves their mechanical response in the elastic region thus indicating significantly stronger structure. All tests were repeated three times with coefficient of variation (CV) 9.67% and 9.45% for four-axis and traditional 3D printing, respectfully.

### 3.3. Thermo-Mechanical Testing of BVS

**Figure 7:**
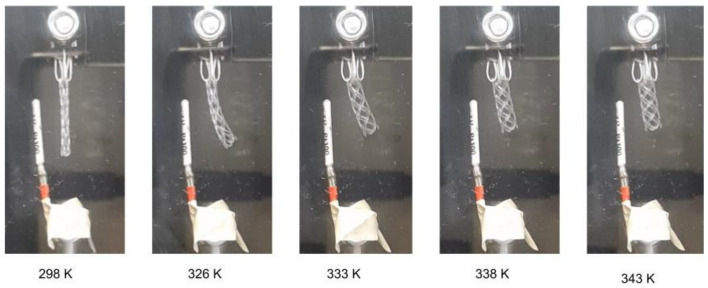
Shape recovery of BVS in the temperature chamber by heating it from the room temperature up to 70 °C.

As previously stated, the shape recovery process is conducted through a thermo-mechanical cycle. Throughout the cycle, load, temperature, and deformation are recorded. In order to comprehend the process of shape recovery, it is essential to consider the three crucial values: temperature, load, and displacement over time. These factors are illustrated in three distinct planes, namely load-displacement, load-temperature, and temperature-displacement. By examining the process through these planes, one can gain a deeper understanding of the thermomechanical ability of PLA BVS. Figure 7 illustrates how the BVS sample behaves as it undergoes the process of free shape recovery in the temperature chamber at temperatures of 25, 50, 60, 65, and 70 °C, respectively.

**Figure 8:**
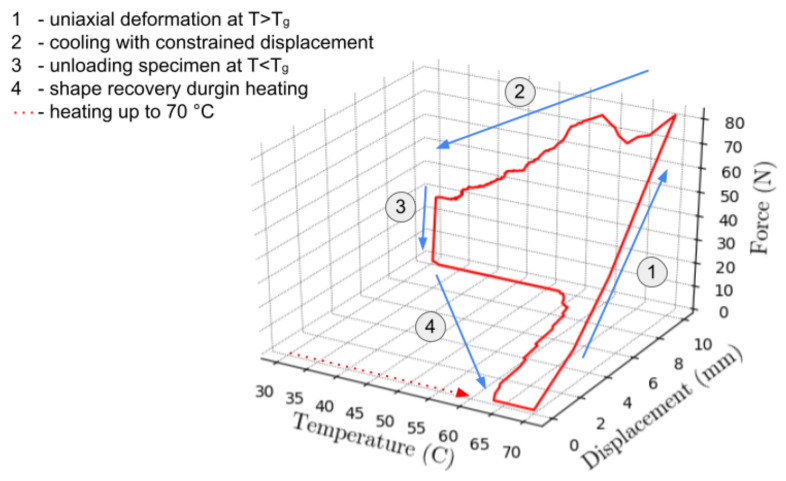
Load-displacement-temperature 3D curve for TMC used in this study.

The results obtained from our samples are presented in Figure 8. The Figure 8 displays a standard TMC with four stages (denoted by the red line in Figure 8):

1. applying uniaxial tension at a temperature above T_g_
2. cooling to a temperature of 30 °C with constrained displacement
3. unloading
4. shape recovery upon reheating over T_g_

**Figure 9:**
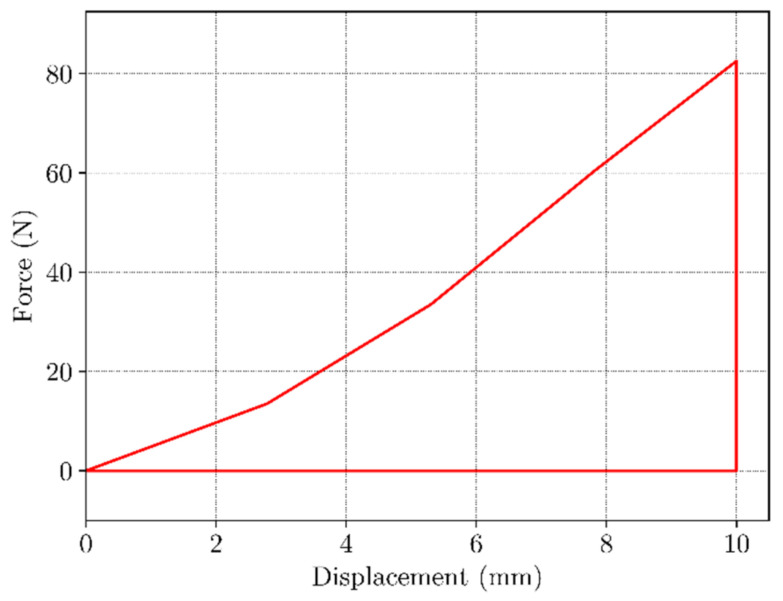
Load-displacement curve under uniaxial tension of BVS at T > T_g_.

**Figure 10:**
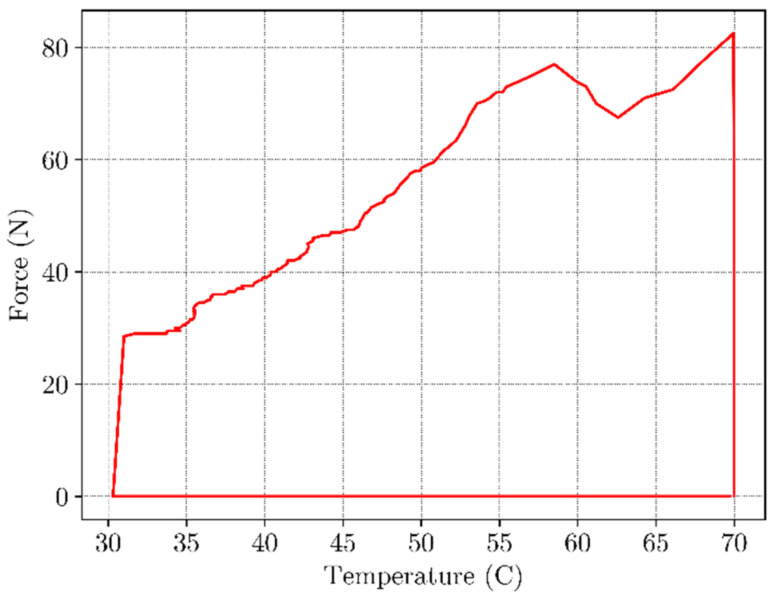
Temperature-load curve under the constrained cooling process.

**Figure 11:**
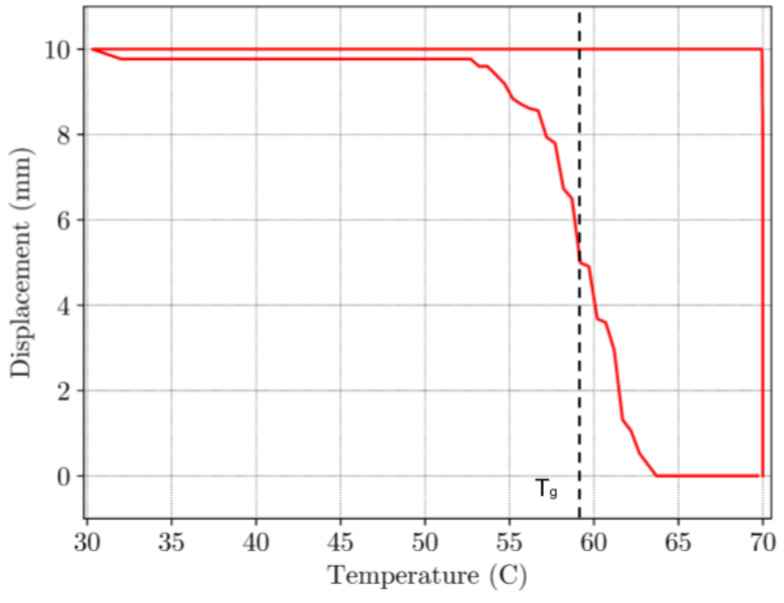
Displacement-temperature curve under the constrained cooling process.

In Figure 9 the load-displacement curve under uniaxial load is shown. As expected, material exhibits a hyperelastic behavior as the deformation occurs at a temperature higher than T_g_, when material is in the flexible “rubbery” state. Strain rate was 0.5 mm/s with total displacement of 10 mm. The maximum load observed at the end of this step was 80 N. The temperature-load curve is given in Figure 10. During constrained displacement cooling process, the load gradually decreases until it reaches final value of 25 N. Once the constrained cooling step is complete, the BVS is unloaded causing the load to decrease to zero. In the final step, the BVS is heated to the temperature of 70 °C transitioning from the low temperature over T_g_ zone up to a temperature where SME is activated. This process is shown in Figure 10. The black dashed line represents the mean value of the temperature between the initial phase of shape recovery and the value when the displacement becomes constant which can be considered as the glass transition temperature value. When temperature is higher than this value SME occurs in a nonlinear manner and requires approx. 120 s for the shape recovery of the sample. Based on the results shown in Figure 11, the sample has recovered its initial shape to approximately 99.7% at the end of the cycle.

**Figure 12:**
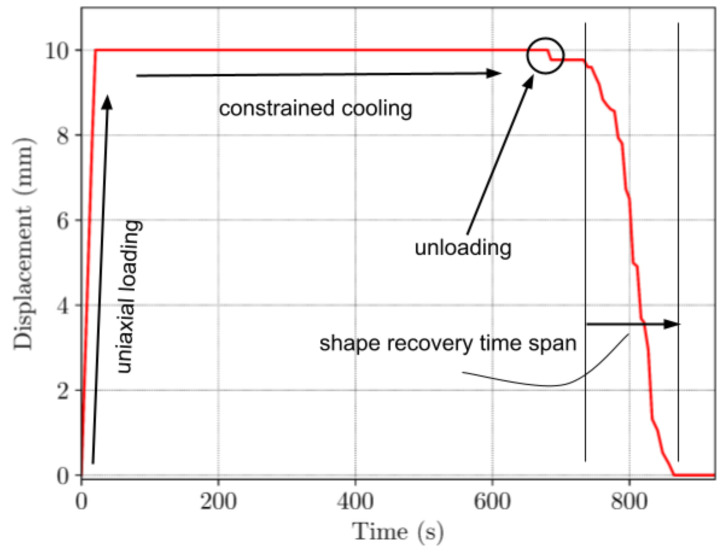
Time-displacement curve for TMC.

Change in the displacement over time for all steps is given in Figure 12. It can be seen on the time scale, that the time necessary for a complete shape recovery was approximately 120 s.

**Figure 13:**
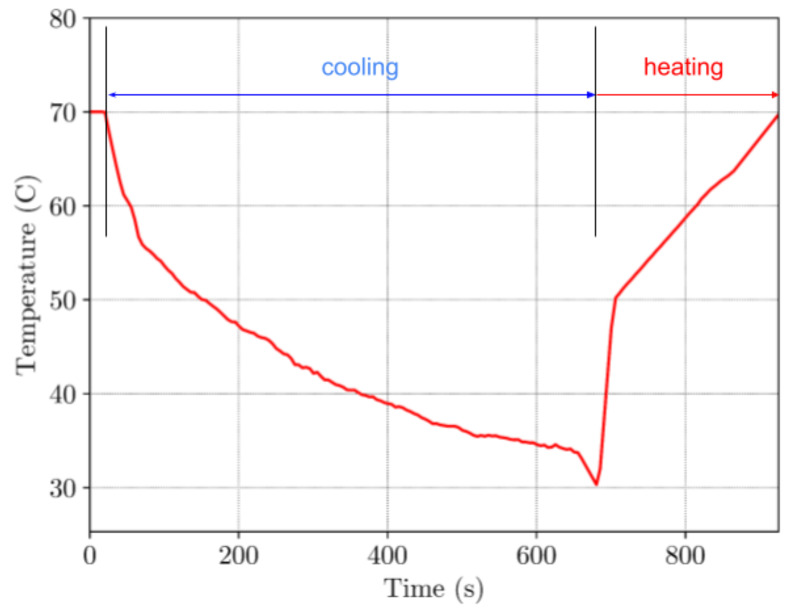
Time-temperature curve for TMC.

Due to the technical limitations of the in-house built temperature chamber, the experiment had to be conducted under specific time intervals. As shown in Figure 13, cooling the chamber down to the temperature of approx. 30 °C (T < T_g_) needs approximately 600 s of programming time. Total time of the full experiment was 900 s.

Each BVS sample was measured after completion of TMC. Height and diameter of the BVSs were compared with values of as printed BVSs. All samples were measured after the final steps given in Figure 12 and Figure 13 and the deformation was calculated according to the formula given in [57]:

(1) $$R_{rec}=\left(1-\varepsilon_{f}\right)\;\cdot \;100\%$$

where $\varepsilon_{f}$ is the final strain value after step 4 of TMC (reheating) which represents the difference between the sample height after the shape recovery and the initial sample height.

### 3.4. Shape Recovery in the Warm Bath

**Figure 14:**
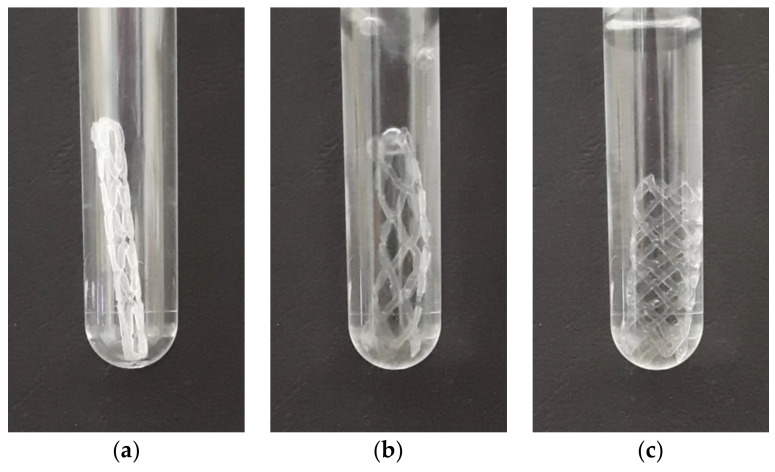
Shape recovery of the BVS in the hot bath, at 70 °C temperature showing: (**a**) deformed shape; (**b**) recovery process; (**c**) fully recovered shape.

The process is different from the previously presented thermo-mechanical cycle because the sample is taken out of the chamber after it has been cooled to a temporary shape. Figure 14 shows the reshaping process in the water bath, as captured by a digital camera. The sample was placed into a see-through container (as shown in Figure 14a) that is heated through the direct contact with a hot bath at 70 °C (as shown in Figure 14b) until it fully recovers its shape (as shown in Figure 14b). The sample was held at this temperature for 60 s to ensure the complete shape recovery process. It is important to note that the temperature remains constant, unlike in the case of previous method of the chamber heating. The reshaping process is notably faster, lasting for only 3 s as opposed to the previous method that needed time of 120 s.

**Figure 15:**
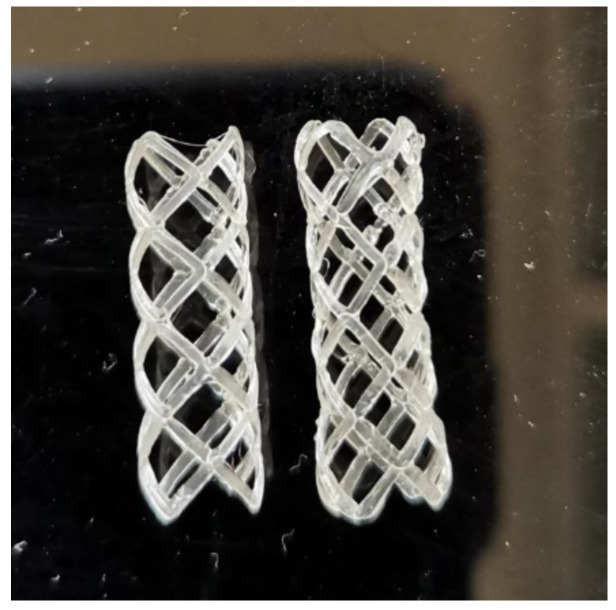
Image of the BVS made by the four-axis printing method: as printed (**left**) and after the shape recovery (**right**).

In Figure 15, two samples made by the new four-axis printing method are shown. As printed sample is shown on the left and the sample on the right was subjected to the testing and thermal shape recovery.

## 4. Conclusions

The objective of this research was to optimize the fabrication procedure of a cylindrical BVS (biodegradable vascular stent) made from SMP (shape-memory polymer) PLA by using a 4D printing technology. The Creality CR-10S5 3D printer has been specifically modified to enable four-axis printing. The newly proposed four-axis printing was realised by direct printing on the rotating drum that enabled enhanced efficiency and precision of the printing process. This printing method of the BVSs resulted in a superior visual and qualitative results in comparison to the traditional layer-by-layer printingof scaffolds. All samples in this study were printed under the same printing conditions—with a 0.4 mm nozzle and at a temperature of 230 °C.

Thermomechanical experiments were realised on the BVS samples produced by four-axis technique. Our findings showed that the samples exhibited remarkable shape memory effects (SME) during a temperature shift from the low temperature to the high temperature zone across the glass transition temperature leading to the full shape recovery.

The mechanical, thermal, and shape recovery characteristics of the printed scaffolds were measured by applying a complete thermo-mechanical cycle, which included load-hold-unload-heating steps. Two techniques to activate SME were used: indirect heating of the samples in a temperature chamber and immersion in a warm bath. In the case of the indirect heating within a chamber, the SME is triggered in the area immediately preceding the T_g_ and intensifies when the temperature is significantly above the T_g_ and material is within the flexible “rubbery” state. The complete recovery of the shape in this scenario requires approximately 100 s. On the other hand, when activated by a hot bath, BVS is immediately exposed to the temperature of 70 °C. The sample required approximately 3 s to fully recover its initial shape in this case.

To effectively simulate SME in PLA, it is crucial for the further research to identify its specific characteristics and define a numerical material model. It is necessary that the material model faithfully reproduces the elastic, viscous, and plastic properties of the SMP PLA, across a broad range of strain rates and temperatures. Integration of the numerical material model into the in-house developed software for the finite element analysis (FEA) will enable simulation of the PLA use in real cases of biomedical applications. It should enable accurate prediction of the effects of PLA during the stent insertion, drug delivery, or tissue growth in antibacterial scaffolds. With this advanced technology, better understanding of the PLA behavior in the human body will be enabled, as well as further development of more effective medical solutions. Further research should be aimed at blend of experiments, simulations, and neural networks that can enhance the speed and accuracy of the simulations through the development of a physically informed neural network (PINN) which begins with new numerical material models. 

## Figures and Tables

**Table 1:** FDM printing parameters.

Printing Technique	Layer Thickness [mm]	Printing Speed [mm/s]	Nozzle Temperature [°C]	Nozzle Diameter [mm]	Bed/Drum Adhesion
**Standard FDM**	0.1	10	230	0.4	Raft
**four-axis FDM**	0.4	10	230	0.4	None

**Table 2:** BVS dimensions.

Length [mm]	Inner Diameter [mm]	Outer Diameter [mm]	Ligament Thickness [mm]	Ligament Width [mm]
20.5	6.2	7	0.5	0.4

## Data Availability

Data is contained within the article.

## References

[1] Palić N., Slavković V., Jovanović Ž., Živić F., Grujović N. (2019). Mechanical Behaviour of Small Load Bearing Structures Fabricated by 3D Printing. Appl. Eng. Lett..

[2] Milenkovic S., Slavkovic V., Fragassa C., Grujovic N., Palic N., Zivic F. (2021). Effect of the Raster Orientation on Strength of the Continuous Fiber Reinforced PVDF/PLA Composites, Fabricated by Hand-Layup and Fused Deposition Modeling. Compos. Struct..

[3] Sam-Daliri O., Ghabezi P., Steinbach J., Flanagan T., Finnegan W., Mitchell S., Harrison N. (2023). Experimental Study on Mechanical Properties of Material Extrusion Additive Manufactured Parts from Recycled Glass Fibre-Reinforced Polypropylene Composite. Compos. Sci. Technol..

[4] (2015). Standard Terminology for Additive Manufacturing—General Principles—Terminology 2015.

[5] Ghabezi P., Flanagan T., Harrison N. (2022). Short Basalt Fibre Reinforced Recycled Polypropylene Filaments for 3D Printing. Mater. Lett..

[6] Sam-Daliri O., Ghabezi P., Flanagan T., Finnegan W., Mitchell S., Harrison N. (2022). Recovery of Particle Reinforced Composite 3D Printing Filament from Recycled Industrial Polypropylene and Glass Fibre Waste. Proc. World Congr. Mech. Chem. Mater. Eng..

[7] Bhattacharjee N., Parra-Cabrera C., Kim Y.T., Kuo A.P., Folch A. (2018). Desktop-Stereolithography 3D-Printing of a Poly(Dimethylsiloxane)-Based Material with Sylgard-184 Properties. Adv. Mater..

[8] Tumbleston J.R., Shirvanyants D., Ermoshkin N., Janusziewicz R., Johnson A.R., Kelly D., Chen K., Pinschmidt R., Rolland J.P., Ermoshkin A. (2015). Continuous Liquid Interface Production of 3D Objects. Science.

[9] Giannatsis J., Vassilakos A., Dedoussis V. (2020). A Heterogeneous Infill Technique for Fused Deposition Modeling. Procedia Manuf..

[10] Lee S.J., Jo H.H., Lim K.S., Lim D., Lee S., Lee J.H., Kim W.D., Jeong M.H., Lim J.Y., Kwon I.K. (2019). Heparin Coating on 3D Printed Poly (l-Lactic Acid) Biodegradable Cardiovascular Stent via Mild Surface Modification Approach for Coronary Artery Implantation. Chem. Eng. J..

[11] Haleem A., Javaid M. (2019). Polyether Ether Ketone (PEEK) and Its 3D Printed Implants Applications in Medical Field: An Overview. Clin. Epidemiol. Glob. Health.

[12] Sun Y., Wang L., Ni Y., Zhang H., Cui X., Li J., Zhu Y., Liu J., Zhang S., Chen Y. (2023). 3D Printing of Thermosets with Diverse Rheological and Functional Applicabilities. Nat. Commun..

[13] Quan H., Zhang T., Xu H., Luo S., Nie J., Zhu X. (2020). Photo-Curing 3D Printing Technique and Its Challenges. Bioact. Mater..

[14] Shehata N., Abdelkareem M.A., Sayed E.T., Egirani D.E., Opukumo A.W. (2022). Smart Materials: The Next Generation. Encyclopedia of Smart Materials.

[15] Qi H.J., Nguyen T.D., Castro F., Yakacki C.M., Shandas R. (2008). Finite Deformation Thermo-Mechanical Behavior of Thermally Induced Shape Memory Polymers. J. Mech. Phys. Solids.

[16] Chu H., Yang W., Sun L., Cai S., Yang R., Liang W., Yu H., Liu L. (2020). 4D Printing: A Review on Recent Progresses. Micromachines.

[17] Bodaghi M., Noroozi R., Zolfagharian A., Fotouhi M., Norouzi S. (2019). 4D Printing Self-Morphing Structures. Materials.

[18] Bodaghi M., Damanpack A.R., Liao W.H. (2016). Self-Expanding/Shrinking Structures by 4D Printing. Smart Mater. Struct..

[19] Li X., Shang J., Wang Z. (2017). Intelligent Materials: A Review of Applications in 4D Printing. Assem. Autom..

[20] Bodaghi M., Damanpack A.R., Liao W.H. (2018). Triple Shape Memory Polymers by 4D Printing. Smart Mater. Struct..

[21] Huang W.M., Song C.L., Fu Y.Q., Wang C.C., Zhao Y., Purnawali H., Lu H.B., Tang C., Ding Z., Zhang J.L. (2013). Shaping Tissue with Shape Memory Materials. Adv. Drug Deliv. Rev..

[22] Yeazel T.R., Becker M.L. (2020). Advancing Toward 3D Printing of Bioresorbable Shape Memory Polymer Stents. Biomacromolecules.

[23] Rahmatabadi D., Soltanmohammadi K., Pahlavani M., Aberoumand M., Soleyman E., Ghasemi I., Baniassadi M., Abrinia K., Bodaghi M., Baghani M. (2023). Shape Memory Performance Assessment of FDM 3D Printed PLA-TPU Composites by Box-Behnken Response Surface Methodology. Int. J. Adv. Manuf. Technol..

[24] Rahmatabadi D., Ghasemi I., Baniassadi M., Abrinia K., Baghani M. (2023). 4D Printing of PLA-TPU Blends: Effect of PLA Concentration, Loading Mode, and Programming Temperature on the Shape Memory Effect. J. Mater. Sci..

[25] Aberoumand M., Soltanmohammadi K., Rahmatabadi D., Soleyman E., Ghasemi I., Baniassadi M., Abrinia K., Bodaghi M., Baghani M. (2023). 4D Printing of Polyvinyl Chloride (PVC): A Detailed Analysis of Microstructure, Programming, and Shape Memory Performance. Macro Mater. Eng..

[26] Yakacki C.M., Shandas R., Lanning C., Rech B., Eckstein A., Gall K. (2007). Unconstrained Recovery Characterization of Shape-Memory Polymer Networks for Cardiovascular Applications. Biomaterials.

[27] Senatov F.S., Niaza K.V., Zadorozhnyy M.Y., Maksimkin A.V., Kaloshkin S.D., Estrin Y.Z. (2016). Mechanical Properties and Shape Memory Effect of 3D-Printed PLA-Based Porous Scaffolds. J. Mech. Behav. Biomed. Mater..

[28] Turek P., Jońca K., Winiarska M. (2023). Evaluation of the Accuracy of the Resection Template and Restorations of the Bone Structures in the Mandible Area Manufactured Using the Additive Technique. Rep. Mech. Eng..

[29] Ge Q., Sakhaei A.H., Lee H., Dunn C.K., Fang N.X., Dunn M.L. (2016). Multimaterial 4D Printing with Tailorable Shape Memory Polymers. Sci. Rep..

[30] Jiang Y., Wang Q. (2016). Highly-Stretchable 3D-Architected Mechanical Metamaterials. Sci. Rep..

[31] Inverardi N., Scalet G., Melocchi A., Uboldi M., Maroni A., Zema L., Gazzaniga A., Auricchio F., Briatico-Vangosa F., Baldi F. (2021). Experimental and Computational Analysis of a Pharmaceutical-Grade Shape Memory Polymer Applied to the Development of Gastroretentive Drug Delivery Systems. J. Mech. Behav. Biomed. Mater..

[32] Farah S., Anderson D.G., Langer R. (2016). Physical and Mechanical Properties of PLA, and Their Functions in Widespread Applications—A Comprehensive Review. Adv. Drug Deliv. Rev..

[33] Jamshidian M., Tehrany E.A., Imran M., Jacquot M., Desobry S. (2010). Poly-Lactic Acid: Production, Applications, Nanocomposites, and Release Studies. Compr. Rev. Food Sci. Food Saf..

[34] Hamad K., Kaseem M., Yang H.W., Deri F., Ko Y.G. (2015). Properties and Medical Applications of Polylactic Acid: A Review. Express Polym. Lett..

[35] Filipovic N., Nikolic D., Isailovic V., Milosevic M., Geroski V., Karanasiou G., Fawdry M., Flanagan A., Fotiadis D., Kojic M. (2021). In Vitro and in Silico Testing of Partially and Fully Bioresorbable Vascular Scaffold. J. Biomech..

[36] Jia H., Gu S.-Y., Chang K. (2018). 3D Printed Self-Expandable Vascular Stents from Biodegradable Shape Memory Polymer. Adv Polym Technol.

[37] Van Manen T., Janbaz S., Jansen K.M.B., Zadpoor A.A. (2021). 4D Printing of Reconfigurable Metamaterials and Devices. Commun. Mater..

[38] Soleyman E., Rahmatabadi D., Soltanmohammadi K., Aberoumand M., Ghasemi I., Abrinia K., Baniassadi M., Wang K., Baghani M. (2022). Shape Memory Performance of PETG 4D Printed Parts under Compression in Cold, Warm, and Hot Programming. Smart Mater. Struct..

[39] Pandini S., Inverardi N., Scalet G., Battini D., Bignotti F., Marconi S., Auricchio F. (2020). Shape Memory Response and Hierarchical Motion Capabilities of 4D Printed Auxetic Structures. Mech. Res. Commun..

[40] Pasini C., Inverardi N., Battini D., Scalet G., Marconi S., Auricchio F., Pandini S. (2022). Experimental Investigation and Modeling of the Temperature Memory Effect in a 4D-Printed Auxetic Structure. Smart Mater. Struct..

[41] Park S.-J., Kang S.-J., Virmani R., Nakano M., Ueda Y. (2012). In-Stent Neoatherosclerosis. J. Am. Coll. Cardiol..

[42] Karanasiou G.S., Papafaklis M.I., Conway C., Michalis L.K., Tzafriri R., Edelman E.R., Fotiadis D.I. (2017). Stents: Biomechanics, Biomaterials, and Insights from Computational Modeling. Ann. Biomed. Eng..

[43] Hou L.-D., Li Z., Pan Y., Sabir M., Zheng Y.-F., Li L. (2016). A Review on Biodegradable Materials for Cardiovascular Stent Application. Front. Mater. Sci..

[44] Nishio S., Kosuga K., Igaki K., Okada M., Kyo E., Tsuji T., Takeuchi E., Inuzuka Y., Takeda S., Hata T. (2012). Long-Term (>10 Years) Clinical Outcomes of First-in-Human Biodegradable Poly-*l*-Lactic Acid Coronary Stents: Igaki-Tamai Stents. Circulation.

[45] Nishio S., Takeda S., Kosuga K., Okada M., Kyo E., Tsuji T., Takeuchi E., Terashima T., Inuzuka Y., Hata T. (2014). Decade of Histological Follow-Up for a Fully Biodegradable Poly-*l*-Lactic Acid Coronary Stent (Igaki-Tamai Stent) in Humans: Are Bioresorbable Scaffolds the Answer?. Circulation.

[46] Tong X., Zhang Z., Fu K., Li Y., Cao B., Wang W., Chen B. (2023). Achieving High Mechanical Properties of Biodegradable Vascular Stents by Four-Axis 3d Printing System and Heat Treatment. Mater. Lett..

[47] Jeżewski M.P., Kubisa M.J., Eyileten C., De Rosa S., Christ G., Lesiak M., Indolfi C., Toma A., Siller-Matula J.M., Postuła M. (2019). Bioresorbable Vascular Scaffolds—Dead End or Still a Rough Diamond?. J. Clin. Med..

[48] Jinnouchi H., Torii S., Sakamoto A., Kolodgie F.D., Virmani R., Finn A.V. (2019). Fully Bioresorbable Vascular Scaffolds: Lessons Learned and Future Directions. Nat. Rev. Cardiol..

[49] Onuma Y., Serruys P.W. (2011). Bioresorbable Scaffold: The Advent of a New Era in Percutaneous Coronary and Peripheral Revascularization?. Circulation.

[50] Foin N., Lee R.D., Torii R., Guitierrez-Chico J.L., Mattesini A., Nijjer S., Sen S., Petraco R., Davies J.E., Di Mario C. (2014). Impact of Stent Strut Design in Metallic Stents and Biodegradable Scaffolds. Int. J. Cardiol..

[51] Regazzoli D., Leone P.P., Colombo A., Latib A. (2017). New Generation Bioresorbable Scaffold Technologies: An Update on Novel Devices and Clinical Results. J. Thorac. Dis..

[52] Milosevic M., Anic M., Nikolic D., Geroski V., Milicevic B., Kojic M., Filipovic N. (2021). Application of in Silico Platform for the Development and Optimization of Fully Bioresorbable Vascular Scaffold Designs. Front. Med. Technol..

[53] Okereke M.I., Khalaj R., Tabriz A.G., Douroumis D. (2021). Development of 3D Printable Bioresorbable Coronary Artery Stents: A Virtual Testing Approach. Mech. Mater..

[54] Nikolic D.D., Filipovic N. (2020). Topological and Parametric Optimization of Stent Design Based on Numerical Methods. Computational Modeling in Bioengineering and Bioinformatics.

[55] Van Kampen K.A., Olaret E., Stancu I.-C., Moroni L., Mota C. (2021). Controllable Four Axis Extrusion-Based Additive Manufacturing System for the Fabrication of Tubular Scaffolds with Tailorable Mechanical Properties. Mater. Sci. Eng. C.

[56] Zivic F., Mitrovic S., Grujovic N., Jovanovic Z., Dzunic D., Milenkovic S. (2021). The Influence of the 3D Printing Infill and Printing Direction on Friction and Wear of Polylactic Acid (PLA) under Rotational Sliding. J. Frict. Wear.

[57] Issabayeva Z., Shishkovsky I. (2023). Prediction of The Mechanical Behavior of Polylactic Acid Parts with Shape Memory Effect Fabricated by FDM. Polymers.

